# Haemodynamic reactions to acute psychological stress and smoking status in a large community sample

**DOI:** 10.1016/j.ijpsycho.2009.04.005

**Published:** 2009-09

**Authors:** Anna C. Phillips, Geoff Der, Kate Hunt, Douglas Carroll

**Affiliations:** a School of Sport and Exercise Sciences, University of Birmingham, Birmingham B15 2TT, England, UK; b MRC Social and Public Health Sciences Unit, University of Glasgow, Glasgow, Scotland, UK

**Keywords:** Acute psychological stress, Blood pressure, Cardiovascular reactivity, Heart rate, Smoking

## Abstract

Exaggerated haemodynamic reactions to acute psychological stress have been implicated in a number of adverse health outcomes. This study examined, in a large community sample, the cross-sectional associations between haemodynamic reactivity and self-reported smoking status. Blood pressure and heart rate were measured at rest and in response to a 3-minute arithmetic stress task. Participants were classified as current, ex-, or non-smokers by their response to a simple prompt. Smokers had significantly smaller SBP and DBP reactions to acute stress than ex- and non-smokers; current and ex-smokers had lower HR reactivity. These effects remained significant following adjustment for a host of variables likely to be associated with reactivity and/or smoking. Although the act of smoking acutely increases haemodynamic activity, the present findings contribute to a growing body of literature showing that smokers have blunted reactivity to mental stress. They also support the hypothesis that blunted reactivity may be characteristic of a range of dependencies. The present results also suggest that smoking status needs to be considered in the design and analysis of stress reactivity studies.

## 1 Introduction

Both the acute and chronic effects of smoking on haemodynamic activity are reasonably well characterised. Blood pressure and heart rate rise immediately after smoking (e.g., Hasenfratz and Battig, 1992; James and Richardson, 1991; Pauli et al., 1993). There is evidence that these acute elevations in haemodynamic activity are due to nicotine: first, they do not occur with sham smoking (Hori et al., 1994), and second, they are also apparent with direct nicotine administration in smokers and non-smokers (Heishman et al., 1993; Perkins et al., 1994). In contrast, a number of epidemiological studies suggest that smokers register lower resting blood pressures than non-smokers (Goldbourt et al., 1997; Green et al., 1986; Handa et al., 1990; Sheffield et al., 1997a). It has been argued that this chronic effect could reflect the temporary abstinence that is usually required with clinical examination (Mann et al., 1991; Parati et al., 1991). However, smokers have also been observed to show attenuated blood pressure during ambulatory monitoring, even though smoking was permitted during the testing period (Green et al., 1991). On the other hand, smokers have been reported to display higher heart rates than non-smokers (Roy et al., 1994; Sheffield et al., 1997a).

The effects of smoking on haemodynamic reactions to psychological stress exposures are much less clear. There would appear to be some consensus regarding the acute effects of smoking on haemodynamic reactivity. A number of studies have found that smoking and psychological stress have additive activating effects (Davis and Matthews, 1990; Macdougall et al., 1988; Ray et al., 1986). Indeed, effects greater than additive have also been reported (Dembroski et al., 1985). Studies comparing smokers and non-smokers in terms of haemodynamic reactivity have found that smokers exhibit: larger reactions to stress, although not for women (Tersman et al., 1991); smaller reactions in women (Girdler et al., 1997; Straneva et al., 2000); smaller reactions for men (Roy et al., 1994); and no difference in reaction (Perkins et al., 1992) compared to non-smokers. In the one large scale study to date, male smokers were found to have smaller systolic blood pressure and heart rate reactions to acute stress, but higher diastolic blood pressure reactions (Sheffield et al., 1997a,b).

Given the lack of agreement among previous studies, we decided to re-visit the issue of smoking status and haemodynamic reactivity. There are also sound theoretical reasons for doing so. The reactivity hypothesis proposes that exaggerated haemodynamic reactions to acute psychological stress are a risk factor for cardiovascular pathology (Lovallo and Gerin, 2003; Schwartz et al., 2003). In support of this, several prospective studies have now shown with reasonable consistency that high reactivity confers an additional risk for a range of cardiovascular outcomes, including high blood pressure, carotid atherosclerosis, carotid intima thickness, and increased left ventricular mass (e.g. Allen et al., 1997; Barnett et al., 1997; Carroll et al., 2003; Kamarck et al., 1997; Lynch et al., 1998; Markovitz et al., 1998; Treiber et al., 2003). Smoking is now widely known to contribute to the aetiology of cardiovascular disease. The questions arises as to whether smokers aggravate or, to an extent, offset that risk by typically exhibiting relatively high or low haemodynamic reactions to psychological stress.

The present analyses of data from a large community sample of men and women allowed us to re-examine the issue of whether the magnitude of haemodynamic reactions to a standard mental stress task, as well as resting haemodynamic activity, was linked to smoking status. The size of the sample afforded sufficient power to extend the analyses beyond exploring simple differences between smokers and non-smokers to investigating variations among smokers, ex-smokers, and participants who had never smoked. In addition, the richness of the database permitted adjustment for a number of potential confounders. Previous studies in this area have either failed to control for, or controlled for very few, likely covariates.

## 2 Materials and methods

### 2.1 Participants

Data were collected as part of the West of Scotland Twenty-07 Study. Participants were all from Glasgow and surrounding areas in Scotland, and have been followed up at regular intervals since the baseline survey in 1987 (Ford et al., 1994). The study was designed to examine the processes that produce and maintain socio-demographic differences in health (Macintyre, 1987). The data reported here are from the third follow-up carried out in 1995. At the third follow-up, cardiovascular reactions to an acute psychological stress task were measured (Carroll et al., 2000, 2003) as was smoking status. Reactivity data were available for 1647 participants, comprising three distinct age cohorts. There were 592 (36%) 24-year olds, 624 (38%) 44-year olds, and 431 (26%) 63-year olds; 890 (54%) were women and 757 (46%) men, and 772 (47%) were from manual and 870(53%) from non-manual occupation households. Household occupational group data were not available for five participants. The sample was almost entirely Caucasian, reflecting the West-of-Scotland population from which it was drawn. Overall mean age at this follow-up was 41.8 (SD = 15.44) years. The exact mean (SD) ages of the young, middle aged, and eldest cohorts were 23.7 (0.56) 44.1 (0.85), and 63.1 (0.67) years, respectively. Mean (SD) body mass index, calculated from measured height and weight was 26.7 (4.26) kg/m^2^. Local ethics committee approval was obtained for each phase of data collection and all the participants provided informed consent at each follow-up.

### 2.2 Apparatus and procedure

Participants were tested in a quiet room in their homes by trained nurses. As part of a structured interview, household occupational group was classified as manual or non-manual from the occupation of the head of household, using the Registrar General's Classification of Occupations (1980). Head of household was usually the man. Participants were classified as never, ex-, or current smokers by their response to a simple question: “do you ever smoke tobacco now? I am thinking of a pipe, cigars, and your own roll-ups as well as cigarettes you might buy.” If the answer was no, they were then asked: “did you ever used to smoke any sort of tobacco?” In addition, current smokers were asked whether they regarded themselves as a very light occasional smoker, a light but regular smoker, a moderate smoker, quite a heavy smoker, or a very heavy smoker. It was also determined whether they were currently taking antihypertensive medication. Participants then undertook an acute psychological stress task: the paced auditory serial addition test (PASAT), which has been shown in numerous studies to reliably perturb the cardiovascular system (Ring et al., 2002; Winzer et al., 1999) and to demonstrate good test-retest reliability (Willemsen et al., 1998). Participants were presented, via audiotape, with a series of single digit numbers and had to add sequential number pairs while retaining the second of the pair in memory for addition to the next number presented, and so on throughout the series. Answers were given orally and, if participants faltered, they were prompted to begin again with the next number pair. The number of correct answers was recorded as a measure of performance. The first sequence of 30 numbers was presented at a rate of one every 4 s, and the second sequence of 30 at one every 2 s. The complete task took three minutes, two minutes for the slower and one minute for the faster sequence. A brief practice was given to ensure that participants understood the demands of the task. Only those who registered a score on the PASAT were included in the analyses. Out of a possible score of 60, the mean score was 40.9 (SD = 9.03).

SBP, DBP and HR were measured by an Omron (model 705CP) semi-automatic sphygmomanometer recommended by the European Society of Hypertension (O'brien et al., 2001). Following the interview, (at least an hour), there was then a formal 5-minute period of relaxed sitting, at the end of which a resting baseline reading of SBP, DBP, and HR was taken. Task instructions were then given, followed by the brief practice. Two further SBP, DBP, and HR readings were taken during the task, the first initiated 20 s into the task (during the slower sequence of numbers), and the second initiated 110 s later (at the same point during the faster sequence). For all readings, the nurses ensured that the participant's elbow and forearm rested comfortably on a table at heart level. The two task readings were averaged and the resting baseline value subsequently subtracted from the resultant average task value to yield reactivity measures for SBP, DBP, and HR for each participant.

### 2.3 Statistical analyses

Repeated measures ANOVAs, comparing baseline and mean task value, were undertaken to confirm that the PASAT perturbed cardiovascular activity. To examine the socio-demographic patterning of smoking, *χ*^2^ was applied. Prior to testing the association between smoking and reactivity, the relationship between smoking and baseline cardiovascular level was examined using ANOVA. Analyses of reactivity (the difference between the mean task and the baseline value) were by ANCOVA, with SBP, DBP, and HR reactivity as the dependent variable, smoking status as the independent variable; and baseline cardiovascular level was entered as a covariate. Where significant effects emerged, further ANCOVAs were undertaken that, in addition to baseline level, adjusted for socio-demographic characteristics (age, sex, occupational group) (Carroll et al., 2000) and other potential confounders previously found to be associated with reactivity in this sample (body mass index, antihypertensive medication status, PASAT performance score, depression) (Carroll et al., 2008, 2007). Slight variations in degrees of freedom reflect occasional missing data for some variables. Significant variations among smoking status groups were followed up by post hoc pairwise comparisons using the Newman-Keuls method. For both ANOVAs and ANCOVAs, partial *η*^2^ is reported as a measure of effect size.

## 3 Results

### 3.1 Smoking status

Five hundred and ninety three (36%) of the sample were current smokers, 338 (21%) were ex-smokers, and 715 (43%) reported never smoking. Data were unavailable for one participant. The socio-demographic breakdown is presented in Table 1. The numbers of both current and never smokers declined with age, whereas the numbers who had given up increased, *χ*^2^ (4) = 80.99, *p* < .001. Participants from manual occupational households were more likely to smoke that those from non-manual households, *χ*^2^ (2) = 51.03, *p* < .001. The sex difference in smoking status was not statistically significant, *χ*^2^ (2) = 5.67, *p* = .06.

### 3.2 Cardiovascular reactivity to the stress task

The PASAT provoked significant increases in SBP, F(1,1646) =1562.32, *p* < .001, *η_p_*^2^ = .487, DBP, F(1,1646) = 1066.62, *p* < .001, *η_p_*^2^ =.393, and HR, F(1,1646) = 1132.96, *p* < .001, *η_p_*^2^ = .408. We have described the socio-demographic effects on reactivity in previous reports (Carroll et al., 2000, 2007) and they are summarized in Table 2. Briefly, HR reactivity declined with age, with the youngest cohort exhibiting higher reactivity than the middle cohort who, in turn, showed higher reactivity than the eldest cohort. HR reactivity was also greater in participants from non-manual occupational status households. The youngest cohort showed smaller SBP and DBP reactions than the other two cohorts and women were less SBP and DBP reactive than men.

### 3.3 Smoking and baseline cardiovascular activity

Baseline cardiovascular levels according to smoking status are presented in Table 3. Ex smokers had higher SBP, F(2,1643) = 9.57, *p* < .001, *η_p_*^2^ = .012, and DBP, F(2,1643) = 8.45, *p* < .001, *η_p_*^2^ = .010, baseline levels than current smokers (*p* < .001 in each case) and those who never smoked (*p* < .001 in each case). There was also a main effect of smoking status on resting HR, F(2,1643) = 4.02, *p* = .02, *η_p_*^2^ = .005; current smokers had higher resting HR than never smokers (*p* = .005). These associations were re-examined in ANCOVAs, adjusting for age cohort, sex, occupational status, body mass index, and whether or not participants were taking antihypertensive medication, which 142 (7%) were. The effects of smoking status on baseline SBP and DBP were abolished, F < 1 in both cases. However, the effect on baseline HR levels survived such adjustment, F(2,1630) = 5.28, *p* = .005, *η_p_*^2^ = .006.

### 3.4 Smoking and cardiovascular reactivity

The summary statistics are presented in Table 3. Adjusting for baseline levels, there were main effects of smoking group on cardiovascular reactivity, for SBP, F(2,1642) = 10.85, *p* < .001, *η_p_*^2^ =.013, DBP, F(2,1642) = 7.02, *p* = .001, *η_p_*^2^ = .008, and HR, F(2,1642) =11.07, *p* < .001, *η_p_*^2^ = .013. Smokers had significantly smaller SBP and DBP reactions than ex smokers (*p* < .001, and *p* = .003, respectively) and those who had never smoked (*p* < .001 in each case). Current smokers also had smaller HR reactions than never (*p* < .001) but not ex smokers.

The analyses were repeated adjusting for, in addition to baseline level, age cohort, sex, occupational status, body mass index, whether or not participants were taking antihypertensive medication, and performance score on the PASAT. The latter is associated with cardiovascular reactivity in this sample (Carroll et al., 2008) and smokers performed more poorly than participants who had never smoked, F(2,1643) = 4.21, *p* = .02, *η_p_*^2^ = .006; the mean (SD) performance scores were 43.0 (9.26), 43.6 (9.29), and 44.5 (9.09), for current, ex, and never smokers, respectively. The effects reported above were still significant following adjustment: F(2,1628) = 10.27, *p* < .001, *η_p_*^2^ = .012, F(2,1628) = 5.32, *p* = .005, *η_p_*^2^ = .006, and F(2,1628) = 8.98, *p* < .001, *η_p_*^2^ = .011, for SBP, DBP, and HR reactivity respectively. We have already reported that reactivity was negatively associated with depressive symptomatology in this sample (Carroll et al., 2007). Symptoms of depression were measured at the third follow up using the Hospital Anxiety and Depression Scale (Zigmond and Snaith, 1983), a well-recognised assessment tool. Depression scores were also higher in smokers than participants who had never smoked, F(2,1605) = 9.55, *p* < .001, *η_p_*^2^ = .012; the mean (SD) scores were 4.0 (2.95), 3.7 (2.87), and 3.3 (2.75), for current, ex, and never smokers, respectively. Accordingly, in another series of ANCOVAs, we adjusted additionally for depression score. Although the smoking status effects were attenuated, they remained statistically significant: F(2,1590) = 6.93, *p* = .001, *η_p_*^2^ = .009, F(2,1590) = 4.89, *p* = .008, *η_p_*^2^ = .006, and F(2,1590) = 3.87, *p* = .02, *η_p_*^2^ = .005, for SBP, DBP, and HR reactivity, respectively. The models with all these covariates accounted for 13%, 12%, and 16% of the variation in SBP, DBP, and HR reactivity, respectively.

### 3.5 Smoking regularity and cardiovascular reactivity

The cardiovascular reactivity of smokers who described themselves as very light occasional smokers (*N* = 87) was compared to that of the rest of the smokers (*N* = 505). There were no differences between groups in blood pressure reactivity. However, the very light occasional smokers (mean = 9.8, SD = 9.58 bpm) tended to exhibit greater HR reactivity than the rest of the smokers (mean = 6.5, SD = 9.15 bpm), F(1,589) = 3.33, *p* = .07, *η_p_*^2^ = .006.

## 4 Discussion

Whereas previous research indicates that the excitatory cardiovascular effects of smoking at the time of exposure add to the haemodynamic impact of acute psychological stress (Davis and Matthews, 1990; Macdougall et al., 1988; Ray et al., 1986), the consequences of being a habitual smoker for reactivity are less clear. Nevertheless, two larger scale studies suggest that, if anything, smokers conventionally exhibit smaller blood pressure and HR reactions to stress than non-smokers (Roy et al., 1994; Sheffield et al., 1997b). The present results from a large community sample provide strong confirmatory evidence. Smokers displayed significantly smaller SBP and DBP reactions to acute stress than ex and non-smokers. Further, current and ex- smokers had lower HR reactivity. The novel inclusion of the ex-smoker category in the present study allows us to determine whether giving up smoking is associated with a similar magnitude of reactivity to that typical of non-smokers; this would certainly appear to be the case for blood pressure, although not HR, reactivity. These associations between smoking status and cardiovascular reactivity remained significant following adjustment for a host of variables likely to be associated with reactivity and/or smoking: baseline cardiovascular level, age cohort, sex, occupational status, body mass index, performance score on the PASAT, whether or not participants were taking antihypertensive medication, and depressive symptoms score. In addition, very light occasional smokers tended to have larger HR reactions to stress than more frequent smokers. Only one previous study, that we know of, considered regularity of smoking; no differences in cardiovascular reactivity were reported between moderate (< 15 cigarettes a day) and heavy smokers (Roy et al., 1994). However, it is likely that the present self-categorisation of very light occasional smokers contained individuals who were much closer to non-smokers than the moderate smokers in the previous study.

The direction of the present association finds common ground with the results of other studies reporting generally blunted physiological adjustments in smokers to a range of challenges. For example, in a study using positron emission tomography to measure myocardial perfusion, smokers were reported to show an impaired increase in perfusion to the cold pressor test relative to non-smokers (Meeder et al., 1996). Further, smokers were found to have a smaller flow velocity response than non-smokers in the posterior cerebral arteries, measured by functional transcranial Doppler, to a visual stimulus challenge (Olah et al., 2008). In addition, smokers have been shown to have reduced density and down-regulated function of beta-adrenergic receptors, which might also contribute to reduced cardiovascular reactivity (Laustiola et al., 1988). These effects have been found to be reversible with smoking cessation (Laustiola et al., 1991). Thus, it would appear that habitual smoking may be characterised by depressed rather than exaggerated sympathetic nervous system reactivity. It is also possible that the associations observed in the present study, to an extent, reflect variations in parasympathetic withdrawal, as smokers have been reported to show a blunted response of the low frequency component of the heart rate variability spectrum to orthostatic stress (Lucini et al., 1996).

The direction of the present associations are also in line with a growing body of evidence that smokers exhibit blunted hypothalamic–pituitary–adrenocortical (HPA) axis stress reactivity. Although the act of smoking and acute doses of nicotine activate the HPA axis (Kirschbaum et al., 1992; Pomerleau et al., 1983), habitual smokers have been found to show diminished salivary and plasma cortisol reactions to psychological stress (Al'absi et al., 2003; Kirschbaum et al., 1993, 1994; Rohleder and Kirschbaum, 2006). In addition, this cortisol hypo-responsiveness has been found to predict relapse among smokers who have recently quit smoking; blunted blood pressure reactivity was also associated with relapse (Al'absi et al., 2005; Al'absi, 2006). Give that haemodynamic and cortisol stress reactivity are strongly correlated (e.g. Cacioppo, 1994), it is perhaps unsurprising that attenuated reactivity in one system is paralleled by the diminished reactions of the other and that blunted reactivity in the two systems may be similarly implicated in smoking relapse. Indeed, it has been argued recently that blunted reactivity, also found in alcoholics and drug abusers, may be a general marker for risk of dependency and addiction (Lovallo, 2006).

Ex-smokers registered higher resting blood pressure than smokers and non-smokers. In previous epidemiological comparisons of smokers and non-smokers, the former are generally reported to have lower blood pressure (Goldbourt et al., 1997; Green et al., 1986; Sheffield et al., 1997b). However, in the present study, group variations in resting blood pressure were abolished following statistical adjustment for a number of covariates. Accordingly, it is possible that findings of relative low resting blood pressure among smokers are artefactual. In contrast, smokers had higher resting HR levels than the other two groups. This result is also not without precedent (Roy et al., 1994; Sheffield et al., 1997b). Further, the effect withstood adjustment for possible confounders.

The present study is not without limitations. First, we measured only blood pressure and HR. It could have proved instructive to have the sort of comprehensive assessment of haemodynamics afforded by impedance cardiography. However, the decision to test participants in their own homes and the size of the sample precluded the use of impedance cardiography. Second, determining causality is impossible from cross-sectional observational data and confounding is always a potential problem (Christenfeld et al., 2004). However, we did adjust statistically for a broad range of possible confounders. Nevertheless, residual confounding as a consequence of poorly measured or un-measured variables cannot be wholly discounted. For example, one very parsimonious explanation for the direction of relationship observed in the present study is that smokers, as a result of temporary abstinence during stress testing (Roy et al., 1994) found it more difficult to concentrate and engaged less with the stress task. This would be reflected in a poorer stress task performance score and current smokers (mean = 43.0, SD = 9.26) did not perform as well as participants who had never smoked (mean = 44.5, SD = 9.09) with ex-smokers (mean = 43.6, SD = 9.29) occupying the mid position, F(2,1643) = 4.21, *p* = .02, *η_p_*^2^ = .005. However, the group differences in performance are small and the associations between smoking status and haemodynamic reactivity remained significant after adjustment for performance score. Third, and relatedly, we had to rely on performance score as our measure of task engagement. Although this seems reasonable, in retrospect it might have proved useful to have included self-report measures of stress task impact. Fourth, it is also possible that nicotine deprivation *per se* as a result of temporary withdrawal may have contributed to blunted reactivity. The present study would undoubtedly have benefited from including a measure of smoking recency. However, blunted cardiac output and heart rate reactivity to acute stressors in female smokers have been observed regardless of whether they were wearing a nicotine replacement patch or placebo patch (Girdler et al., 1997). In addition, cardiovascular reactivity has been compared among non-smokers, smokers who abstained from smoking, and smokers who continued to smoke at their usual rate; smokers, irrespective of their assigned condition, showed attenuated blood pressure reactions to psychological stress (Al'absi et al., 2003). Thus, we think it unlikely that any short-term abstinence in the present study would have affected reactivity dramatically. Finally, our main measure of smoking behaviour was crude to say the least. For example, it has been argued that in terms of the total exposure to the toxic smoke components of tobacco, the way in which cigarettes are smoked may be as important as whether people smoke Jarvis and Russell (1980). In the present study no account was taken of the extent of inhalation. However, subjective measures of inhalation have proved unsatisfactory (Stepney, 1982) and most previous studies, including those on smoking and haemodynamic reactivity, have relied on simple taxonomies of the sort used here. Although smoking is sometimes underestimated in self-reports, particularly among ex-smokers (Lewis et al., 2003), there is evidence that smokers' reports can be reasonably reliable and agree with objective measures such as CO exhalation (Mak et al., 2005). Nevertheless, it would have strengthened the study had more detailed information regarding duration and intensity of smoking behaviour been available, as well as biochemical measures to verify smoking behaviour.

In conclusion, compared to non-smokers and/or ex-smokers, current smokers exhibited blunted haemodynamic reactions to acute psychological stress. The effects, although attenuated, were still significant following adjustment for a wide range of candidate confounders. These findings may have both theoretical and methodological ramifications. First, it would appear that smokers' increased risk for cardiovascular disease is not a function of increased reactivity. Second, the direction of the associations observed is consistent with the notion that blunted stress reactivity in smokers may be common to both the sympathetic nervous system and the HPA axis. Third, it resonates with the idea that blunted reactivity may be characteristic of a range of dependencies. Further studies, however, will be required to confirm this sort of speculation. Fourth, smoking status is rarely if ever mentioned as a variable that needs to be considered when designing stress reactivity studies or in analysing the data from them. As others have noted (Roy et al., 1994), it may be necessary to adjust for smoking status or to treat smokers and non-smokers separately when analysing the effects of other psychosocial or behavioural variables on haemodynamic reactions to stress.

## Figures and Tables

**Table 1:** Smoking(%) by age cohort, sex, and household occupational status.

		Current	Ex-	Never
Age Cohort	Youngest	239 (40)	58 (10)	294 (50)
	Middle	218 (35)	140 (22)	266 (43)
	Eldest	136 (32)	140 (32)	155 (36)
Sex	Male	287 (38)	165 (22)	305 (40)
	Female	306 (34)	173 (20)	410 (46)
Occupational Group	Manual	343 (45)	155 (20)	273 (35)
	Non-manual	248 (29)	181 (21)	441 (50)

**Table 2:** Mean (SD) SBP, DBP, and HR during baseline and the stress task by age cohort, sex, and occupational status.

Group		SBP		DBP		HR	
		Baseline	Task	Baseline	Task	Baseline	Task
Age Cohort	Youngest	120.0 (15.07)	130.1 (15.93)	73.4 (10.08)	80.2 (10.27)	67.5 (11.00)	77.5 (12.74)
	Middle	127.1 (18.08)	139.4 (18.75)	80.6 (11.13)	87.7 (11.30)	66.7 (11.17)	74.5 (12.17)
	Eldest	144.4 (21.68)	156.7 (22.80)	83.8 (11.17)	90.8 (13.23)	65.7 (9.92)	71.7 (11.15)
Sex	Male	134.7 (18.25)	147.5 (12.22)	81.2 (11.18)	88.4 (11.73)	64.7 (10.43)	73.4 (12.19)
	Female	124.3 (21.07)	134.7 (21.06)	76.8 (11.56)	85.6 (12.34)	68.4 (10.84)	76.0 (12.32)
Occupational Group	Manual	130.5 (21.44)	141.7 (22.17)	79.3 (11.93)	85.9 (12.56)	67.0 (11.26)	74.0 (12.19)
	Non-manual	127.8 (19.58)	139.6 (21.16)	78.4 (11.29)	85.7 (12.06)	66.5 (10.40)	75.6 (12.39)

**Table 3:** Mean (SD) SBP, DBP, and HR baseline and reactivity by smoking status.

		Current	Ex	Never
SBP	Baseline	128.2 (20.30)	133.4 (21.41)	127.8 (19.98)
	Reactivity	9.8 (11.41)	12.4 (11.57)	12.4 (11.97)
DBP	Baseline	78.3 (11.70)	81.2 (11.74)	78.2 (11.36)
	Reactivity	6.0 (8.09)	7.1 (8.27)	7.6 (9.15)
HR	Baseline	67.7 (11.18)	66.5 (10.93)	66.0 (10.38)
	Reactivity	6.9 (9.28)	7.1 (8.75)	9.5 (10.43)
